# Generative adversarial networks for imputing missing data for big data clinical research

**DOI:** 10.1186/s12874-021-01272-3

**Published:** 2021-04-20

**Authors:** Weinan Dong, Daniel Yee Tak Fong, Jin-sun Yoon, Eric Yuk Fai Wan, Laura Elizabeth Bedford, Eric Ho Man Tang, Cindy Lo Kuen Lam

**Affiliations:** 1 Department of Family Medicine and Primary Care, Faculty of Medicine, University of Hong Kong, Hong Kong, Hong Kong SAR, China; 2 School of Nursing, Faculty of Medicine, University of Hong Kong, Hong Kong, Hong Kong SAR, China; 3 Electrical and Computer Engineering Department, University of California, Los Angeles, CA USA; 4 Department of Pharmacology and Pharmacy, Faculty of Medicine, University of Hong Kong, Hong Kong, Hong Kong SAR, China

**Keywords:** Generative adversarial network, Missing data imputation, Machine learning, Clinical research, Big data

## Abstract

**Background:**

Missing data is a pervasive problem in clinical research. Generative adversarial imputation nets (GAIN), a novel machine learning data imputation approach, has the potential to substitute missing data accurately and efficiently but has not yet been evaluated in empirical big clinical datasets.

**Objectives:**

This study aimed to evaluate the accuracy of GAIN in imputing missing values in large real-world clinical datasets with mixed-type variables. The computation efficiency of GAIN was also evaluated. The performance of GAIN was compared with other commonly used methods, MICE and missForest.

**Methods:**

Two real world clinical datasets were used. The first was that of a cohort study on the long-term outcomes of patients with diabetes (50,000 complete cases), and the second was of a cohort study on the effectiveness of a risk assessment and management programme for patients with hypertension (10,000 complete cases). Missing data (missing at random) to independent variables were simulated at different missingness rates (20, 50%). The normalized root mean square error (NRMSE) between imputed values and real values for continuous variables and the proportion of falsely classified (PFC) for categorical variables were used to measure imputation accuracy. Computation time per imputation for each method was recorded. The differences in accuracy of different imputation methods were compared using ANOVA or non-parametric test.

**Results:**

Both missForest and GAIN were more accurate than MICE. GAIN showed similar accuracy as missForest when the simulated missingness rate was 20%, but was more accurate when the simulated missingness rate was 50%. GAIN was the most accurate for the imputation of skewed continuous and imbalanced categorical variables at both missingness rates. GAIN had a much higher computation speed (32 min on PC) comparing to that of missForest (1300 min) when the sample size is 50,000.

**Conclusion:**

GAIN showed better accuracy as an imputation method for missing data in large real-world clinical datasets compared to MICE and missForest, and was more resistant to high missingness rate (50%). The high computation speed is an added advantage of GAIN in big clinical data research. It holds potential as an accurate and efficient method for missing data imputation in future big data clinical research.

**Trial registration:**

ClinicalTrials.gov ID: NCT03299010; Unique Protocol ID: HKUCTR-2232

**Supplementary Information:**

The online version contains supplementary material available at 10.1186/s12874-021-01272-3.

## Background

Missing data is a pervasive problem in big data research, clinical trials and epidemiological studies [1]. There are a number of reasons that could account for missing data, such as non-response to questionnaires, study participants lost to follow up, omission of data entry, failure of equipment, or incomplete or lost records [2, 3]. Mere exclusion of cases with missing data from analysis may lead to biased inference, reduced statistical power and generalisability of results [4, 5]. According to missingness assumptions, the problem of missing data can be classified into three categories, including missing completely at random (MCAR), missing at random (MAR), and missing not at random (MNAR) [6–8]. In general, the majority of the missing data in medical research are assumed to be MAR [9]. In contrast to MCAR, where there are no systematic differences between the missing and observed values, with MAR data, there will be differences between missing and observed values but these differences can be explained by other observed data [10–12].

Multiple imputation by chained equations (MICE) is the most commonly used statistical procedure for handling missing data [5], particularly for data that are MAR [13]. MICE is widely available in many statistical software including SPSS, STATA and R. Although it is important to note that MICE may lead to biased results because, by default it uses predictive mean matching (pmm) and logistic regression (LR), which are limited in the ability to handle non-linear relationships and interactions between variables [14]. A mean to overcome non-linearity is through random forest, an ensemble machine learning algorithm of multi-classification or decision tree regression [15]. Stekhoven et al. have developed a method ‘missForest’ (based on random forest) to impute missing values in mixed-type datasets [16]. Subsequent studies have shown that missForest outperformed MICE in both simulated and real world datasets [17]. However, a drawback of missForest is that its long computation time, limiting its practicality in big data research.

Generative adversarial network (GAN), an unsupervised algorithm, is a popular machine learning method that has been widely applied in both data generation [18] and image processing [19]. Generative adversarial imputation nets (GAIN), which is based on GAN, was recently developed and found to outperform other methods in terms of imputation accuracy in substituting MCAR data in five open-source datasets [20]. However, the accuracy of GAIN for imputing MAR data and of mixed-type variables, both of which are common in medical research remains unclear.

The main aim of this study was to evaluate the accuracy of GAIN in imputing missing values in real-world clinical datasets with mixed-type variables. Further, this study also aimed to examine the computation efficiency of GAIN as well as compare its performance with those of MICE and missForest. It is anticipated that the results will inform other researchers on the choice of missing data imputation methods in big clinical data research.

## Methods

### Study setting and datasets

Two large real world clinical datasets from two longitudinal cohort studies on primary care patients with chronic diseases were used. The first dataset was that of a study on the prediction of complications and mortality among a cohort of 141,516 patients with diabetes [21]. A total of 14 (out of 21) independent baseline variables had missing data, of which 12 variables had a missingness rate of less than 20%. Overall, the proportion of missing data ranged from 0.50% (systolic blood pressure) to 48.99% (urine albumin to creatinine ratio [Urine ACR]). Urine ACR showed the highest proportion of missing data as it was not routinely collected in Hong Kong primary care prior to 2010. We selected 50,000 subjects without any missing values for these 21 variables (15 continuous predictors and six categorical predictors) at baseline and seven dependent outcome variables measuring various complications of diabetes and mortality.

The second dataset was that of a cohort study evaluating the effectiveness of a risk assessment and management programme for patients with hypertension [22]. We identified 10 independent variables, including five continuous variables and five categorical variables, for inclusion in the analyses. In the original dataset, the data missingness rate for these 10 variables ranged from 1.5 to 26%. A total of 10,000 subjects without any missing values for these 10 variables were randomly selected. The data were extracted together with the data for the two outcome variables (cardiovascular diseases [CVD] and mortality) in order to replicate the imputation analyses and strengthen the generalizability of the results from the first dataset.

For easy reference, the first dataset is referred as the ‘DM-data’ and the second is referred as the ‘HT-data’. The description of the characteristics for these two datasets can be found in Supplementary Tables 1 & 2.

Institutional Review Board of the University of Hong Kong—the Hospital Authority Hong Kong West Cluster (reference number: UW 15–258) approved this study and usage of data. Individualized informed consent is not required. All methods on the datasets were carried out in accordance with relevant guidelines and regulations.

### Missing data simulation

For both DM-data and HT-data, data ‘missing at random’ (MAR) was simulated at different missingness rates (20 and 50%) to create the datasets for the imputation testing [17, 23]. The missingness was introduced to independent variables following Bernoulli distributions based on linear combination of dependent variables (fully-observed). At each missingness rate, ten different incomplete datasets were generated using different randomised linear combination parameters. We did not simulate missing values in the dependent variables, although they were incorporated in the imputation process as auxiliary variables [24].

### Imputation procedures with GAIN

A number of improvements were applied to the basic GAIN construction built by Yoon et al. [20] to optimize the model. First, the random noise was substituted by the mean value of each variable so as to reach the optimal solution faster. Batch normalization with gradient descent optimizer was also used to allow a larger learning rate. Combination of the loss of continuous and categorical variables with separate weights (α and β) was used to deal with a dataset with mixed types of variables. A greedy search strategy was adopted to seek the best combination of hyper-parameters. This strategy was adopted due to the large number of hyper-parameters to be tuned in the GAIN training process, including k, *p*_*hint*_, α, β, number of iterations, number of hidden layers, number of neurons in each layer, activation functions, learning rate and optimizer. The code is available at Github (https://github.com/dongdongdongdwn/GAIN-Dovey) and the optimal hyper-parameters are presented in the Supplementary Table 3. The brief imputation procedures with GAIN are presented in Algorithm 1.

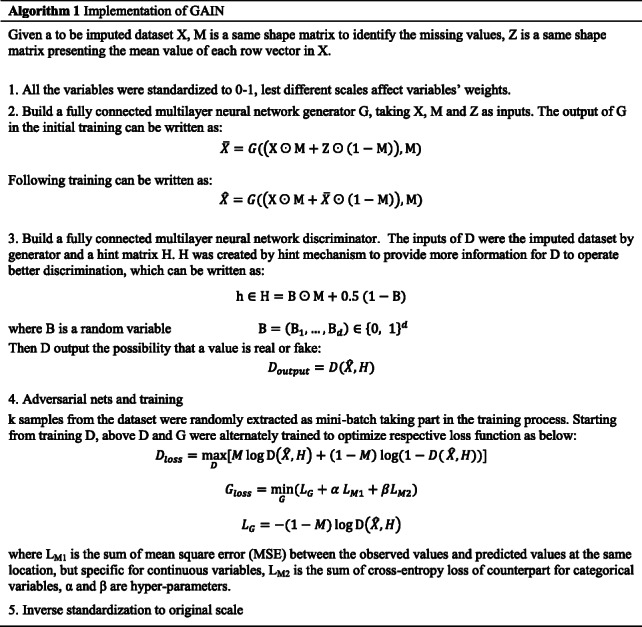



### MICE and missForest

Imputation by MICE and missForest were carried out by standard procedures [16, 24] with R package *mice v3.6.0* and *missForest*. The imputation model of MICE was specified as predictive mean matching (pmm) and logistic regression (LR) as default, respectively, for continuous variables and categorical variables. The iteration number was set to 10. For missForest, the number of trees was set to 20, and the number of variables randomly sampled at each split was set to ${d}^{\frac{1}{2}}$ (sqrt dimensionality). The max-iterations number of missForest was set to 10. The iteration numbers of MICE and missForest were determined based on preliminary experiments to ensure they could achieve the best performance (as shown in Supplementary Fig. 1).

### Outcome measures and data analysis

Accuracy was measured by imputation error, defined as the difference between the imputed values and real values. It was assessed by normalized root mean square error (NRMSE) for continuous variables and proportion of falsely classified (PFC) subjects for categorical variables. NRMSE and PFC were defined as follows:

$$ \mathrm{NRMSE}=\frac{\sqrt{\frac{1}{N}{\sum}_{i=1}^N{\left(\hat{x_i}-{x}_i\right)}^2}}{\frac{\sum_{i=1}^N{x}_i}{N.}} $$

$$ \mathrm{PFC}=1-\frac{N_{correct}}{N} $$

where $\hat{x_i}$ is the imputed value and *x*_*i*_ is the original value in continuous variables, *N*_*correct*_ is the total number of correctly classified values in categorical variables.

For each simulated incomplete dataset, the imputation was repeated 100 times using each method. The mean NRMSE for each continuous variable was calculated by averaging the NRMSE obtained from the 100 imputations. The mean PFC was calculated by averaging the PFC obtained in each imputation for categorical variables. NRMSE and PFC were treated as continuous variables in the comparative analysis, and their distributions were tested by Shapiro-Wilk normality test. Correspondingly, the differences in mean NRMSE or PFC among methods were tested by one-way ANOVA or non-parametric test.

Density plots and bar plots were used to illustrate the imputation differences among methods, for representative continuous variables and categorical variables respectively. For the DM-data, systolic blood pressure (SBP), fasting glucose, hypertension history and smoking status were selected to represent normal distributed continuous variables, skewed continuous variables, balanced categorical variables and imbalanced categorical variables, respectively. Likewise, age, total cholesterol to high-density lipoprotein (TC/HDL) ratio, sex and lipid lowering drugs usage were selected as the representative variables for the HT-data.

For DM-data with 5000 to 50,000 subjects, the computation time of each method to complete an imputation process on a personal computer (PC) and high performance computing (HPC) device was recorded and plotted for comparison. The relevant machine configuration of the PC and HPC can be found in Supplementary Table 3.

Missing data simulation, MICE, missForest and comparison were operated in R 3.5.1. GAIN was developed with Python 3.5. The level of significance for all statistical tests was set as 0.05.

## Results

### Experiments on DM-data

Table 1 presents the imputation errors (NRMSE and PFC for continuous and categorical variables, respectively) of different imputation methods at missingness rates of 20 and 50%. Overall, GAIN and missForest were superior to MICE for both continuous and categorical variables, irrespective of the missingness rates (*p* < 0.001). When the missingness rate was 20%, GAIN was superior to missForest with lower imputation errors (*p* < 0.05) for highly skewed (skewness> 4) continuous variables (e.g., creatinine, fasting glucose, urine ACR) and highly imbalanced categorical variables (proportion of minority class was close to or lower than 10%, e.g., lipid lowering drug usage, DM treatment). MissForest showed better accuracy for some normally distributed continuous variables (e.g., age, SBP, DBP) and some relatively balanced categorical variables (e.g., sex, hypertension history) (*p* < 0.05). GAIN and missForest showed similar accuracy for the remaining variables (*p* > 0.05). However, GAIN was superior to missForest for the majority of variables when the missingness rate increased to 50% (*p* < 0.05). No statistically significant differences were observed between GAIN and missForest for the less skewed continuous variables (e.g., age, SBP, LDL-C) and relatively balanced categorical variables (e.g., sex, hypertension history).

**Table 1:** Imputation errors of different methods in DM-data

	Skewness or proportion of minority class	Missingness rate = 20%			Missingness rate = 50%		
		MICE	missForest	GAIN	MICE	missForest	GAIN
***Continuous variables***							
Age, years	−0.106	0.078 ± 0.002	0.060 ± 0.001 ^a,b^	0.069 ± 0.002 ^a^	0.137 ± 0.002	0.107 ± 0.001 ^a^	0.111 ± 0.002 ^a^
SBP, mmHg	0.316	0.052 ± 0.001	0.041 ± 0.002 ^a,b^	0.048 ± 0.002 ^a^	0.099 ± 0.001	0.082 ± 0.002 ^a^	0.080 ± 0.002 ^a^
DBP, mmHg	0.154	0.070 ± 0.002	0.052 ± 0.002 ^a,b^	0.056 ± 0.002 ^a^	0.120 ± 0.002	0.094 ± 0.001 ^a^	0.090 ± 0.001 ^a,c^
LDL-C, mmol/L	0.379	0.095 ± 0.003	0.075 ± 0.003 ^a,b^	0.089 ± 0.003 ^a^	0.208 ± 0.003	0.163 ± 0.004 ^a^	0.161 ± 0.004 ^a^
BMI, kg/m2	0.813	0.064 ± 0.003	0.048 ± 0.002 ^a^	0.048 ± 0.003 ^a^	0.120 ± 0.004	0.095 ± 0.002 ^a^	0.090 ± 0.005 ^a,c^
Waist, cm	0.299	0.047 ± 0.002	0.036 ± 0.001 ^a^	0.036 ± 0.001 ^a^	0.088 ± 0.002	0.069 ± 0.001 ^a^	0.067 ± 0.003 ^a^
TC, mmol/L	0.564	0.065 ± 0.003	0.050 ± 0.002 ^a,b^	0.055 ± 0.003 ^a^	0.140 ± 0.003	0.110 ± 0.003 ^a^	0.102 ± 0.004 ^a,c^
DM duration, years	−1.167	0.284 ± 0.007	0.206 ± 0.006 ^a^	0.190 ± 0.006 ^a,c^	0.451 ± 0.01	0.340 ± 0.006 ^a^	0.304 ± 0.012 ^a,c^
eGFR, ml/min/1.73 m2	1.368	0.089 ± 0.010	0.057 ± 0.004 ^a,b^	0.087 ± 0.006	0.195 ± 0.012	0.159 ± 0.012 ^a^	0.146 ± 0.015 ^a,c^
HbA1c, %	1.557	0.106 ± 0.004	0.077 ± 0.002 ^a^	0.078 ± 0.004 ^a^	0.177 ± 0.007	0.138 ± 0.004 ^a^	0.125 ± 0.004 ^a,c^
HDL-C, mmol/L	2.729	0.132 ± 0.016	0.111 ± 0.014 ^a^	0.115 ± 0.011 ^a^	0.251 ± 0.011	0.197 ± 0.014 ^a^	0.184 ± 0.015 ^a,c^
TG, mmol/L	3.932	0.287 ± 0.027	0.251 ± 0.022 ^a^	0.266 ± 0.027	0.610 ± 0.027	0.486 ± 0.023 ^a^	0.444 ± 0.026 ^a,c^
Creatinine, μmol/L	4.128	0.093 ± 0.011	0.089 ± 0.016	0.068 ± 0.015 ^a,c^	0.218 ± 0.013	0.177 ± 0.019 ^a^	0.169 ± 0.015 ^a,c^
Fasting glucose, mmol/L	4.681	0.178 ± 0.043	0.121 ± 0.008 ^a^	0.118 ± 0.007 ^a,c^	0.277 ± 0.024	0.214 ± 0.011 ^a^	0.195 ± 0.010 ^a,c^
Urine ACR, mg/mmol	11.450	2.509 ± 0.441	1.728 ± 0.307 ^a^	1.554 ± 0.266 ^a,c^	3.843 ± 0.405	2.987 ± 0.240 ^a^	2.690 ± 0.258 ^a,c^
***Categorical variables***							
Lipid drug usage	8.50%	0.162 ± 0.013	0.093 ± 0.010 ^a^	0.083 ± 0.009 ^a,c^	0.159 ± 0.006	0.090 ± 0.004 ^a^	0.079 ± 0.005 ^a,c^
Smoker	10.57%	0.176 ± 0.014	0.113 ± 0.010 ^a^	0.094 ± 0.009 ^a,c^	0.182 ± 0.013	0.122 ± 0.007 ^a^	0.097 ± 0.008 ^a,c^
DM treatment	10.50%	0.179 ± 0.013	0.115 ± 0.009 ^a^	0.095 ± 0.009 ^a,c^	0.187 ± 0.011	0.120 ± 0.006 ^a^	0.096 ± 0.003 ^a,c^
Hypertension drug usage	29.68%	0.318 ± 0.020	0.256 ± 0.015 ^a^	0.267 ± 0.016 ^a^	0.345 ± 0.01	0.281 ± 0.011 ^a^	0.274 ± 0.013 ^a,c^
Sex	45.93%	0.205 ± 0.020	0.126 ± 0.009 ^a,b^	0.235 ± 0.027	0.353 ± 0.011	0.276 ± 0.01 ^a^	0.287 ± 0.014 ^a^
Hypertension history	47.190%	0.122 ± 0.011	0.077 ± 0.008 ^a,b^	0.129 ± 0.040	0.255 ± 0.012	0.201 ± 0.019 ^a^	0.215 ± 0.017 ^a^

Notes

*SBP* Systolic Blood Pressure, *DBP* Diastolic Blood Pressure, *LDL-C* Low Density Lipoprotein-Cholesterol, *BMI* Body Mass Index, *TC* Total Cholesterol, *eGFR* Estimated Glomerular Filtration, *HbA1c* Hemoglobin A1c, *HDL-C* High Density Lipoprotein-Cholesterol, *TG* Triglyceride, *Urine ACR* Urine Albumin to Creatinine Ratio

Since NRMSE and PFC both followed normal distribution (Shapiro-Wilk normality test *p* value > 0.05), imputation errors of different methods were compared using one-way ANOVA. If *p* < 0.05, paired methods were compared using independent sample t-test

^a^The mean imputation error is significantly lower than that of MICE (*p* < 0.05)

^b^The mean imputation error is significantly lower than that of GAIN (*p* < 0.05)

^c^The mean imputation error is significantly lower than that of missForest (*p* < 0.05)

### Experiments on HT-data

The imputation errors in the HT-data of different methods are presented in Table 2. The findings were similar to those found in the DM-data. Overall, GAIN and missForest outperformed MICE for both missingness rates (20 and 50%) irrespective of the type of variables. When the missingness rate was 20%, GAIN was superior to missForest for more skewed continuous variables (e.g., SBP, TC/HDL-C ratio, hospital admission times) and more imbalanced categorical variables (e.g., smoking, hypertensive drugs, lipid lowering drugs). If the missingness rate increased to 50%, GAIN was more accurate than missForest for the majority of the variables (*p* < 0.05).

**Table 2:** Imputation errors of different methods in HT-data

	Skewness or proportion of minority class	MICE	missForest	GAIN
**Missingness rate = 20%**				
***Continuous variables***				
Age, years	−0.018	0.063 ± 0.002	0.049 ± 0.001 ^a,b^	0.057 ± 0.004 ^a^
SBP	0.492	0.075 ± 0.001	0.058 ± 0.000 ^a^	0.048 ± 0.000 ^a,c^
Charlson index	0.146	0.154 ± 0.002	0.121 ± 0.001 ^a,b^	0.144 ± 0.003 ^a^
TC/HDL-C ratio	3.139	0.175 ± 0.003	0.137 ± 0.001 ^a^	0.115 ± 0.001 ^a,c^
Hospital admission times	7.037	2.379 ± 0.069	1.885 ± 0.042 ^a^	1.752 ± 0.141 ^a,c^
***Categorical variables***				
Smoking	7.45%	0.133 ± 0.007	0.123 ± 0.003 ^a^	0.098 ± 0.010 ^a,c^
Hypertensive drugs	8.10%	0.149 ± 0.006	0.126 ± 0.003 ^a^	0.098 ± 0.002 ^a,c^
Lipid Lowering drugs	9.99%	0.173 ± 0.007	0.159 ± 0.003 ^a^	0.129 ± 0.006 ^a,c^
Overweight	37.89%	0.433 ± 0.01	0.400 ± 0.005 ^a^	0.359 ± 0.003 ^a,c^
Sex	41.21%	0.448 ± 0.019	0.412 ± 0.004 ^a^	0.405 ± 0.022 ^a^
**Missingness rate = 50%**				
***Continuous variables***				
Age, years	−0.018	0.129 ± 0.002	0.102 ± 0.001 ^a^	0.094 ± 0.007 ^a,c^
SBP	0.492	0.115 ± 0.001	0.095 ± 0.001 ^a^	0.080 ± 0.002 ^a^
Charlson index	0.146	0.295 ± 0.001	0.239 ± 0.002 ^a^	0.241 ± 0.009 ^a^
TC/HDL-C ratio	3.139	0.279 ± 0.004	0.235 ± 0.003 ^a^	0.183 ± 0.002 ^a,c^
Hospital admission times	7.037	3.766 ± 0.12	3.199 ± 0.057 ^a^	3.004 ± 0.246 ^a,c^
***Categorical variables***				
Smoking	7.45%	0.335 ± 0.006	0.277 ± 0.015 ^a^	0.267 ± 0.012 ^a,c^
Hypertensive drugs	8.10%	0.368 ± 0.014	0.305 ± 0.004 ^a^	0.276 ± 0.005 ^a,c^
Lipid Lowering drugs	9.99%	0.441 ± 0.015	0.319 ± 0.006 ^a^	0.304 ± 0.009 ^a,c^
Overweight	37.89%	1.135 ± 0.018	1.029 ± 0.019 ^a^	0.850 ± 0.020 ^a,c^
Sex	41.21%	1.149 ± 0.02	1.050 ± 0.013 ^a^	1.007 ± 0.055 ^a^

Notes

*SBP* Systolic Blood Pressure, *TC* Total Cholesterol, *HDL-C* High-Density Lipoprotein Cholesterol

Since NRMSE and PFC both followed normal distribution (Shapiro-Wilk normality test *p* value > 0.05), imputation errors of different methods were compared using one-way ANOVA. If *p* < 0.05, paired methods were compared using independent sample t-test;

^a^The mean imputation error is significantly lower than that of MICE (*p* < 0.05)

^b^The mean imputation error is significantly lower than that of GAIN (*p* < 0.05)

^c^The mean imputation error is significantly lower than that of missForest (*p* < 0.05)

**Fig. 1:**
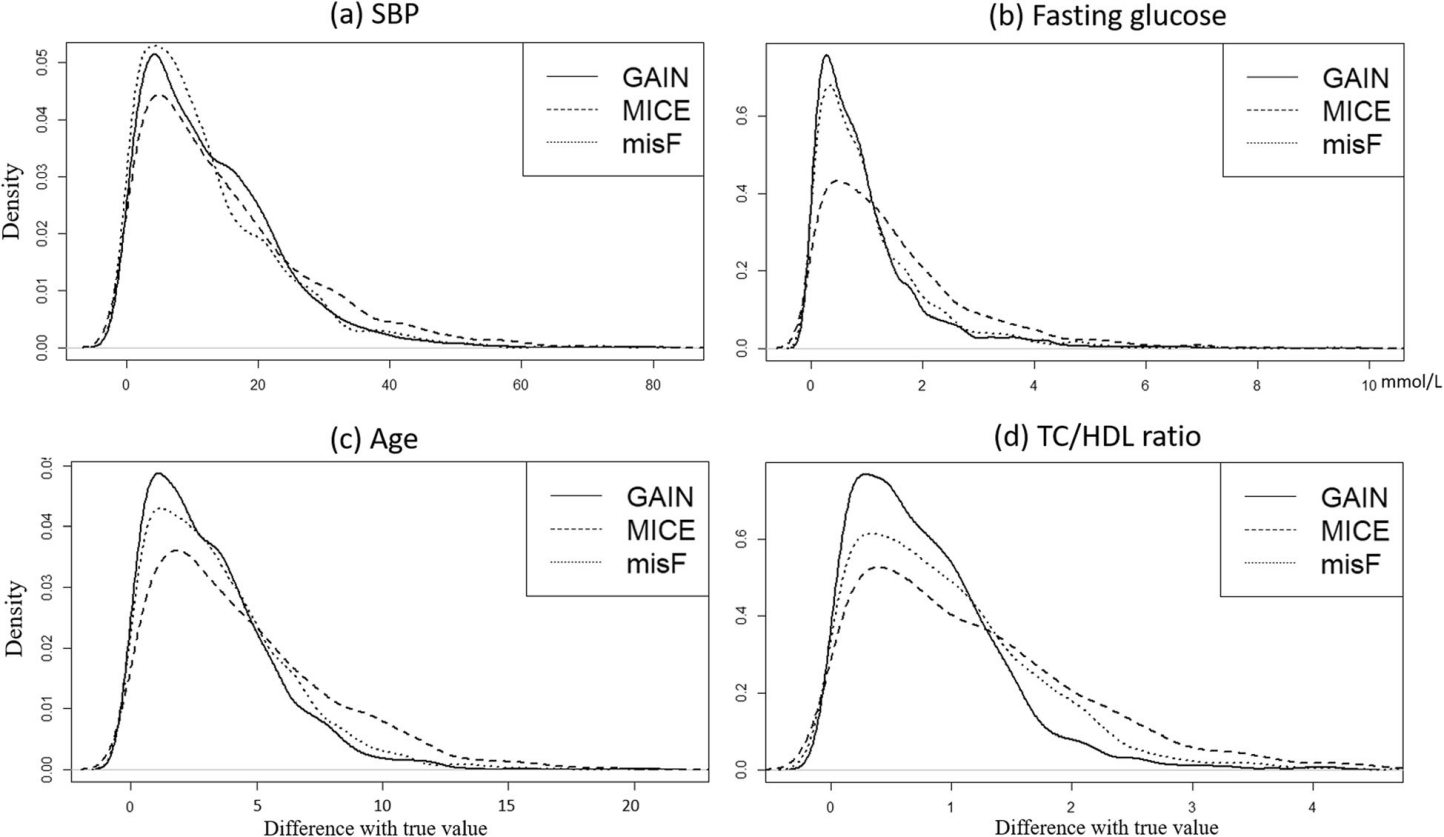
Density plots displaying the distribution of the absolute difference between imputed values and true values on continuous variables by different methods (missingness rate = 50%). (Note: **a** and **b** are representative continuous variables in DM-data, **c** and **d** are representative continuous variables in HT-data)

To illustrate the differences of the imputation errors among methods, density plots and bar plots were used to visualize the representative variables at 50% missingness rate. Density plots, showing the distribution of the absolute difference between imputed values and real values of continuous variables, are presented in Fig. 1. The absolute differences between real values and values generated by GAIN were more close to 0 and concentrated, indicating good accuracy. MICE tended to have a broader distribution of errors and a higher density of greater errors. The differences in the patterns among different methods were more noticeable on data that were skewed (e.g. fasting glucose, TC/HDL ratio).

**Fig. 2:**
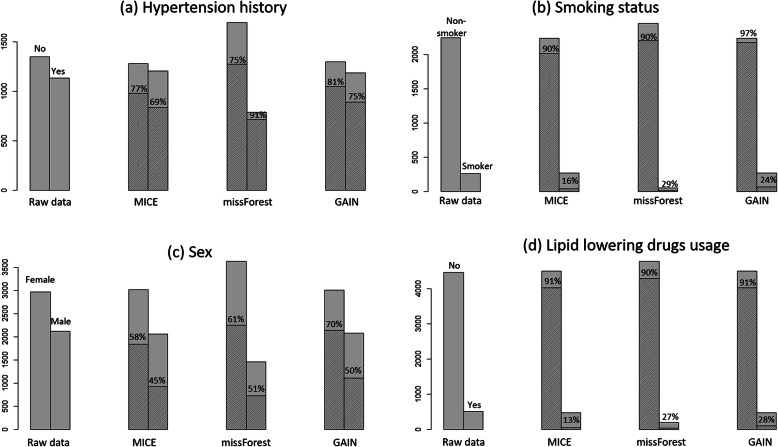
Bar plots displaying the distribution of imputed allocation of categorical variables by different methods (missingness rate = 50%). (Note: **a** and **b** are representative continuous variables in DM-data, **c** and **d** are representative continuous variables in HT-data; Shaded areas indicate the proportion that correctly imputed in each category by each method)

The bar plots illustrate the distribution of imputed values and the correct proportion in each category (Fig. 2). The imputed values of MICE and GAIN showed the same distribution as the original data, while missForest generated a higher proportion of the majority group but a lower proportion of the minority group. Meanwhile, for both balanced (i.e. sex, hypertension history) and imbalanced categorical variables (i.e. smoking, lipid lowering drugs usage), GAIN imputation resulted in a more accurate allocation to the minority group when compared to the other two methods.

### Computation time

**Fig. 3:**
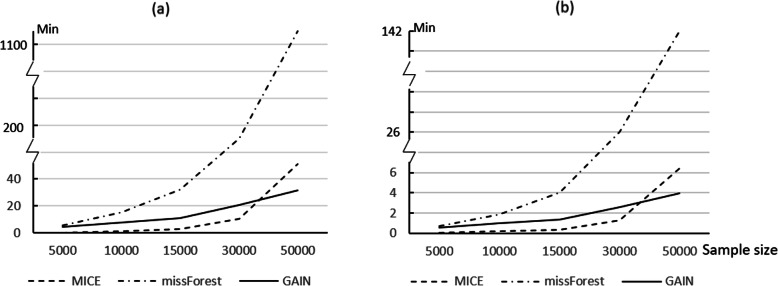
Computation time of one imputation process by each method on DM-data. **a** Computation time on PC; **b** Computation time on HPC

The computation time of one imputation process on the DM-data by each method using PC and HPC for different sample sizes are presented in Fig. 3. MICE was the fastest for small sample sizes (up to 30,000 subjects) and GAIN was the fastest for the larger sample (50,000 subjects). MissForest showed much longer computation times for all sample sizes compared to the other two methods. The computation time of missForest increased exponentially with increasing sample size.

## Discussion

Missing data are inevitable in medical research and it is important that appropriate methods are used to solve this problem in order to make full use of the data and get unbiased inference. This study has introduced a novel imputation method, GAIN, and demonstrated its imputation accuracy and efficiency outperformed two commonly used methods (MICE and missForest). The major strength of this study was the use of two large real-world clinical datasets with mixed-type variables. To the best of our knowledge, this was the first study to evaluate the application of GAIN for the imputation of missing clinical data with mixed type variables.

Overall, GAIN showed similar imputation accuracy as missForest when the missingness rate was relatively low (20%) but performed better than missForest when the missingness rate was higher (50%). GAIN also had better accuracy for imputing skewed continuous variables and imbalanced categorical variables. Furthermore, the imputation time of GAIN increased only slightly with increasing sample size, making it the most efficient method for performing big data analytics on a sample size of more than 30,000.

### Imputation performance and data characteristic

These findings matched those observed in an earlier study where GAIN outperformed other imputation methods on a cancer dataset in which all variables were continuous [20]. It is important to recognise that the imputations of mixed-type variables are challenging but essential for clinical research [10]. Our results provide preliminary evidence that GAIN is a suitable method for the imputation of missing clinical data with mixed type of variables, particularly those with highly skewed and imbalanced data.

The results of this study also showed that, despite MICE being commonly used, there is still room for its improvement [14, 15]. As can be seen from the density plots, the default setting of MICE (pmm) replicated some observed extreme values to seek for the same distribution as the observed data, however, these extreme values might be far from the real values and lead to inaccuracy. On the other hand, missForest and GAIN, through machine learning, are more “moderate” and produced credible values, which are closer to the mean level of the observed data, yielding more accurate imputation results.

### Imputation performance and missingness rate

It is recognized that data with a higher missing proportion are likely to increase further inference bias. There is no consensus on the maximum missing data rate that would allow for substitution by imputation since it is determined by various factors, including the missingness assumption, participation of auxiliary variables, data quality and also imputation methods [25]. In medical research and clinical trials, the rule of thumb for an acceptable missingness rate is 20% or less [26, 27], but much higher rates are commonly observed in real practice. For example, as shown in the two large real-world clinical datasets in this study, the data missingness rates of some variables were nearly 50%. In order to explore how the imputation accuracy would be affected by the data missingness rate, we evaluated the three methods on simulated data with missingness rates of 20 and 50%. It was found that GAIN was more resistant to the effects of a higher missingness rate. This is because the imputation power of GAIN depend not only on observed values but also on the feedback from the discriminator. GAIN therefore has the potential to accept a higher threshold of data missingness rate and maximize the use of research data.

### Computation time

In addition to the measures on accuracy, this study also recorded the computation time as a performance indicator. Computation time cannot be neglected, especially with the large datasets in many cohort studies. GAIN stands out in its efficiency by virtue of its unique mechanism in which the number of parameters is relatively independent of the sample size.

Multiple imputation (MI) is recommended to avoid the uncertainty of single imputation. However, it will increase the computation time. In general, if MI is adopted, the imputation times (m) is at least 5 with some researchers using 10 or more [8]. MissForest will take approximately 8 days (200 h) of PC computation time to impute the missing data with a sample size of 50,000 with multiple imputation of 10 times. The computation time will also lengthen exponentially as the sample size increases. The utilization of HPC and parallel processing may save some time but may not be feasible in many settings.

### Further implication for practice

There is no one best procedure to solve the problem of missing data in medical research. Indeed, the selected method will depend on the missingness assumption as well as auxiliary variables that could explain why the data is missing [28]. For example, complete case analysis might be preferable over MI in some situations [20]. Based on our findings, we would suggest taking into consideration missingness rate, variable distribution, and the expected computation time when choosing the appropriate imputation method. In addition, the use of more than one imputation method and sensitivity analysis could improve the reliability of the results.

### Limitation

This study had a number of limitations. First and foremost, this study had only focused on the imputation accuracy but not post-imputation statistical inference effectiveness of different imputation methods. The goal of missing data imputation is to obtain statistically valid inferences from incomplete data rather than to re-create the true data. Van Buuren has pointed out that imputation is not prediction, and the method that best recovers the true data might be nonsensical or contain severe flaws [8]. Further studies should be conducted to evaluate these imputation methods with respect to post-imputation statistical inferences. Second, a missingness rate of more than 50% was not simulated in this study as some researchers have suggested that a missingness rate of more than 50% is not acceptable for clinical studies [25]. Third, the variables included in this study were cross-sectional data, hence the results may not be generalizable to missing data problem in longitudinal studies with repeated observations.

## Conclusion

Overall, when compared to MICE and missForest, GAIN showed better accuracy in the imputation of missing data in large real world clinical datasets, particularly for imbalanced and skewed data, and when the missingness rate was high (50%). GAIN also has outstanding computation speed in handling large samples (greater than 30,000 subjects) and holds potential as an accurate and efficient method for missing data imputation in future big data clinical research.

## Supplementary Information

**Additional file 1: Supplementary Table 1.** Characteristics Description of DM-data (*N* = 50,000). **Supplementary Table 2.** Characteristics Description of HT-data (*N* = 10,000). **Supplementary Table 3.** Computing devices and model hyper-parameters. **Supplementary Figure 1.** Performance of MICE and missForest at different iteration numbers.

## Data Availability

The data that support the findings of this study are available from Hong Kong Hospital Authority (HKHA) but restrictions apply to the availability of these data, which were used under license for the current study, and so are not publicly available. Data are however available from the authors upon reasonable request and with permission of HKHA.

## Abbreviations

DM: Diabetes mellitus
GAIN: Generative adversarial imputation nets
GAN: Generative adversarial network
HDL: High-density lipoprotein
HPC: High performance computing
HT: Hypertension
LR: Logistic regression
MAR: Missing at random
MCAR: Missing completely at random
MICE: Multiple imputation by chained equations
MNAR: Missing not at random
NRMSE: Normalized root mean square error
PC: Personal computer
PFC: Proportion of falsely classified
pmm: Predictive mean matching
SBP: Systolic blood pressure
TC: Total cholesterol
