# Supplementary material for: Generative adversarial networks for imputing missing data for big data clinical research
Source: BMC Med Res Methodol. 2021 Apr 20;21:78. doi: 10.1186/s12874-021-01272-3 (PMC8059005; doi:10.1186/s12874-021-01272-3)
Supplement: Supplementary file 1 — Additional file 1: Supplementary Table 1. Characteristics Description of DM-data (N = 50,000). Supplementary Table 2. Characteristics Description of HT-data (N = 10,000). Supplementary Table 3. Computing devices and model hyper-parameters. Supplementary Figure 1. Performance of MICE and missForest at different iteration numbers. [file 12874_2021_1272_MOESM1_ESM.docx]

**Generative Adversarial Networks for Imputing Missing Data for Big Data Clinical Research**

Weinan DONG^1^, Daniel Yee Tak Fong^2^, Jin-sun Yoon^3^, Eric Yuk Fai Wan ^*1,4^, Laura Elizabeth Bedford^1^, Eric Ho Man Tang^1^, Cindy Lo Kuen Lam^1^

^1^ Department of Family Medicine and Primary Care, Faculty of Medicine, University of Hong Kong, Hong Kong SAR, China

^2^ School of Nursing, Faculty of Medicine, University of Hong Kong, Hong Kong SAR, China

^3^ Electrical and Computer Engineering Department, University of California, Los Angeles, CA, USA

^4^ Department of Pharmacology and Pharmacy, Faculty of Medicine, University of Hong Kong, Hong Kong SAR, China

***Correspondence:**

Dr. Yuk Fai Eric, WAN.

yfwan@hku.hk

Fax: (852)2814-7475, Tel: (852)2518-5657, Address: 3/F, Ap Lei Chau Clinic, 161 Main Street, Ap Lei Chau, Hong Kong SAR, China

**List of Supplementary Materials:**

Supplementary Table 1. Characteristics Description of DM-data;

Supplementary Table 2. Characteristics Description of HT-data;

Supplementary Table 3. Computing devices and model hyper-parameters;

Supplementary Figure 1. Performance of MICE and missForest at different iteration numbers

Supplementary Table 1. Characteristics Description of DM-data (N= 50,000)

|  | Mean (SD) | Range |  |  |  | %(n) |
| --- | --- | --- | --- | --- | --- | --- |
| ***Continuous variables*** | |  |  | ***Categorical variables*** | | |
| Age, years | 63.229 (11.069) | 18 - 104 |  | Sex | Male | 54.07%(28495) |
| SBP, mmHg | 138.198 (17.852) | 53 - 232 |  |  | Female | 45.93%(24203) |
| DBP, mmHg | 74.824 (10.616) | 33 - 141 |  | Smoking status | Smoker | 10.57%(5570) |
| LDL-C, mmol/L | 3.107 (0.846) | 0 - 9.7 |  | Lipid lowering drugs usage | Yes | 8.5%(4482) |
| BMI, kg/m^2^ | 25.365 (3.839) | 10.16 - 59 |  | DM treatment(oral DM drug/insulin) | Yes | 89.5%(47154) |
| Waist, cm | 88.834 (9.827) | 31 - 152 |  | Hypertension history | Yes | 47.19%(24870) |
| TC, mmol/L | 5.081 (0.968) | 0.3 - 18.7 |  | Hypertension drugs usage | Yes | 70.32%(37058) |
| TG, mmol/L | 1.7 (1.168) | 0.02 - 20 |  | ***Dependent variables*** | |  |
| HDL-C, mmol/L | 1.223 (0.344) | 0.04 - 11.1 |  | CHD | Yes | 9.87%(5201) |
| Creatinine, μmol/L | 80.282 (24.249) | 12 - 836 |  | Stroke | Yes | 10.22%(5387) |
| Urine ACR, mg/mmol | 9.104 (38.503) | 0 - 1099.762 |  | HF | Yes | 5.62%(2964) |
| HbA1c, % | 7.569 (1.474) | 4 - 19.6 |  | ESRD | Yes | 4.71%(2483) |
| FBG, mmol/L | 7.843 (2.459) | 0.4 - 94 |  | First CVD | Yes | 20.8%(10960) |
| DM duration, years | 1.547 (0.729) | 0 - 2.917 |  | Death | Yes | 15.42%(8125) |
| eGFR, ml/min/.173m^2^ | 101.98 (26.906) | 5 - 991 |  | Composite events | Yes | 29.95%(15781) |

SBP: systolic blood pressure; DBP: diastolic blood pressure; LDL-C: low-density lipoprotein; BMI: body mass index; TC: total cholesterol; HDL-C: high-density lipoprotein; Urine ACR: urinary albumin creatinine ratio; HbA1c: hemoglobin A1c; FBG: fasting blood glucose; eGFR: estimated glomerular filtration rate; CHD: coronary heart disease; HF: heart failure; ESRD: end-stage renal disease; CVD: cardiovascular disease.

Supplementary Table 2. Characteristics Description of HT-data (N= 10,000)

|  | Mean (SD) | Range |  |  | %(n) |
| --- | --- | --- | --- | --- | --- |
| ***Continuous variables*** |  |  | ***Categorical variables*** |  |  |
| Age, years | 65.809 (11.553) | 26 - 100 | Overweight | Yes | 62.11% (6211) |
| SBP, mmHg | 136.18 (15.782) | 80 - 221 | Hypertension drugs usage | Yes | 8.10% (810) |
| TC/HDL-C ratio | 3.968 (1.139) | 1.0 - 13.6 | Lipid lowering drugs usage | Yes | 9.99% (999) |
| Charlson Index | 3.096 (1.217) | 0 - 11 | Sex | Male | 41.21% (4121) |
| Hospital visit times | 0.142 (0.524) | 0 - 10 | Smoking status | Yes | 7.45% (745) |
|  |  |  | ***Dependent variables*** |  |  |
|  |  |  | CVD death | Yes | 2.70% (270) |
|  |  |  | All-cause mortality | Yes | 8.22% (822) |

SBP: systolic blood pressure; TC: total cholesterol; HDL-C: high-density lipoprotein; CVD: cardiovascular disease.

Notes: Charlson Index was calculated excluding HIV and DM related complications.

Supplementary Table 3. Computing devices and model hyper-parameters

| Configuration of computation devices |
| --- |
| PC: Processor: Intel(R) Core(TM) i7-7700 CPU @ 3.60GHz; Installed memory (RAM): 32.0GB  HPC: 104 compute nodes outfitted with two 10-core Intel Xeon E5-2600 v3 (Haswell) processors and 96 GB physical memory. |
| Hyper-parameters of GAIN |
| Mini batch number = 200; p_hint = 0.1; alpha = 10; beta = 0.5; interation number = 3000; number of hidden layers = 2; neuron number in hidden layer 1 = 28; neuron number in hidden layer 2 = 28; activation function = tanh; optimizer = SGD; training rate of G = 0.5; training rate of D = 0.2 |

Note:

The computations were performed using research computing facilities offered by Information Technology Services, the University of Hong Kong.

Supplementary Figure 1. Performance of MICE and missForest at different iteration numbers


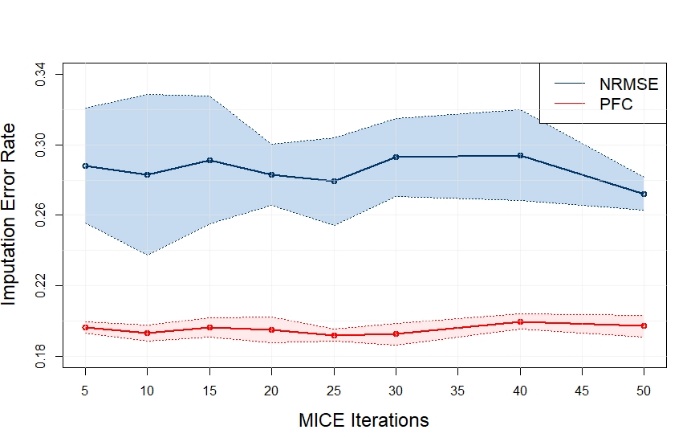

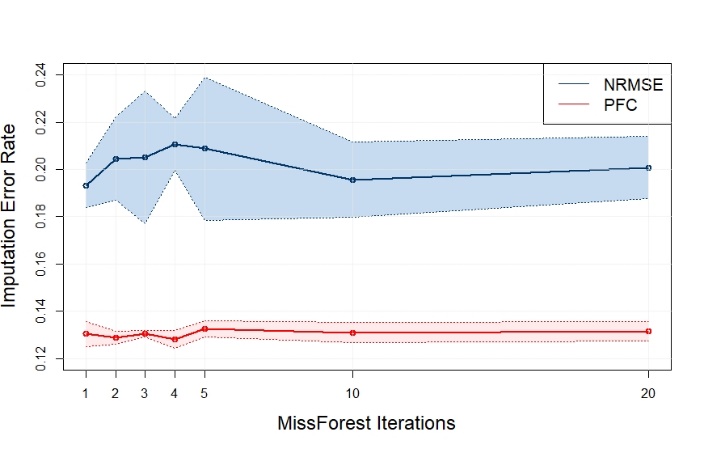


Figure 1. Performance of MICE and missForest at different iteration numbers

(Note: With DM-data, missing data (missingness rate 20%) were introduced and then imputed using MICE and missForest with iterations from 5 to 50 and 1 to 20, respectively. Blue line and surface represent total NRMSE for continuous variables and corresponding 95%CI; red line and its surface represent total PFC for categorical variables and corresponding 95%CI. For both MICE and missForest, the imputation errors (NRMSE, PFC) did not decline significantly with increasing iteration numbers (ANOVA, *P*>0.05). Accordingly, we set the iteration number to 10 for both MICE and missForest in our main analyses, which is also in line with the instructions of these two methods.)
